# Trichinosis1Read before the Pennsylvania State Veterinary Medical Association, September 3, 1895.

**Published:** 1896-02

**Authors:** Otto Noack

**Affiliations:** Reading, Pa.


### TRICHINOSIS.[1]

By OTTO NOACK, V. S.,
READING, PA.

*History.* Not only a very painful but also a very dangerous disease is trichinosis. Tracing back history and description we find it to be known to the profession as far back as 1821. Undoubtedly it was in existence long before that time, but was classified and treated as rheumatism, gastro-enteritis, and other diseases with similar symptoms. The following cases will show it: It was recorded by Tuengel, 1851, that during an epidemic at Hamburg, a man inflicted with the prevalent epidemic was treated and recovered. Dying ten years later, his body was dissected, and the muscles found to contain any amount of trichinæ. Zenkel, of Dresden, reports, during an epidemic of typhus, 1860, he dissected bodies having died from the prevailing epidemic, but found no trace of typhus. Instead, he

[1] Read before the Pennsylvania State Veterinary Medical Association, September 3, 1895.

discovered the muscles filled with innumerable trichinæ. In reports from 1821 we hear of a description of calcified bodies found in dissecting a corpse, though most authors deny the identity with trichinæ. Hilton is also credited with having found trichinæ in 1834 in a case at London Hospital, but thought that these calcified capsules had been formed by the existence of small cysticerces. Again, others claim that James Paget was the first to discover the worm in 1835. Be that as it may, this is certain, the great English naturalist, R. Owen, was the first to describe the parasite in 1835, and name it *trichina spiralis*, still known by that name the world over. His description of the worm is unsatisfactory and not properly classified. A better description is found somewhat later in the same year by Farre. In the fourth decade trichinæ were found to exist in various animals—rats, cats; also found to exist in hogs by Leidy, of Philadelphia, in 1846. Hence the liability of trichinæ in man can be proven. But Virchow was the first to give a definite and perfect description of trichinæ. In 1860, in a more complete description, the difference between the trichinæ in the intestines and the muscles was stated. Thirteen years later, Gerlach, one of the greatest veterinarians, gives us a good and definite description of trichinæ and trichinosis in animals, especially in hogs.

*Description of Trichinæ.* Biology: The trichina (*trichina spiralis*) belongs to the class of nematodes. The trichina has two stages of development, namely, the intestinal and muscle trichina, the latter being the larva of the first. There is a difference of sex, the female growing larger than the male; the former being 3 to 4 mm. in length and 30 to 40 $\mu$ in diameter, and can be recognized by the naked eye by a faint yellowish-white line. The male is about 1 to 1.5 mm. long and 25 to 30 $\mu$ in diameter. The tail-end has two digital appendages. The genital tube forms with the end of the intestine a reversible cloaca. The female has intestinal and genital organs, ovaries, uterus, and vulva. The external genital duct is situated very far front, between the first and second quarter of the body length. The anterior part is conical-shaped, mouth unarmed, the posterior thin and slightly curved. The intestinal trichinæ is generally found in the small, rarely in the large, intestine. The muscle-trichinæ is always found in the sarcolemma of the striated muscle-fibres, with the exception of the heart. However, Virchow, Leukart, Mueller, and others quote a few cases, having found them in very small numbers in the muscle of the heart.

*History of Development.* Whenever partaking of meat that contains muscle-trichinæ, the capsules are dissolved by the gastric juice and the worms become free. After the lapse of two days the worm attains its full growth, according to the statement of Kratz. It is then that the most dangerous part occurs. Copulation takes place, the female becomes pregnant; after five days the first embryo are born. The parturition continues for several weeks, during which time the mother-worm gives birth to more than a thousand embryo. The death of the mother-worm occurs generally after the lapse of from five to eight weeks, passing out with the feces. The young intestinal trichinæ perforate the tissue of the intestines at once, passing through the peritoneal and mucous structure of lymphatic tissue and also the bloodvessels, locating themselves in the sarcolemma, generally near the tendinous insertion of muscles, covering themselves with their chitina, which produces a calcified capsule within two years' time (Gerlach). The elliptic form of the capsule can be recognized by the naked eye as a very fine white spot. Each capsule generally contains one, seldom more worms. According to some authors the worm can attain an age of twenty-five years (Klopsch). This is very doubtful. It takes a temperature of about 160° F. to kill the worm, showing what indestructible vitality the worm has.

*How the Disease is Acquired.* The only possible way for mankind to acquire the disease is by eating raw or only undercooked pork. The worm in the capsule achieves its active life, being freed from the capsule by the gastric juice of the stomach. How the hogs get trichinæ cannot be positively said. Some authors claim that rats infect the hogs; others, say *vice versa.* Leisering claims if all rats were put out of existence the trichinæ would cease to exist. Of the many instances given we find more in favor of this theory than opposed to it, Leukart and Gerlach being in favor of it. Undoubtedly this is certain, trichinæ can only be obtained by eating any food containing trichinæ. Dogs and cats can only be infected by eating mice, rats, and infected meat. The latter become infected, as Fuerstenberg claims, through human excrements. A whole herd can become infected when one hog has trichinæ, as experiments made by Gerlach and Guenther have shown.

*Prevalence of Trichinæ.* Besides man trichinæ prevail in the following animals: Hedge-hog, pole-cat, marten, marmot, badger, hog, cat, rat, and fox. According to Gerlach, horses, rabbits, and guinea-pigs can be infected by feeding it directly, but this cannot be said with certainty of sheep and calves. There are no probabilities of the disease dying out, as it is carried in so many various ways, existing in so many classes of animals throughout the world. Rats eating one another also cause it.

*Symptoms of Trichinosis.* It is very difficult, almost impossible, for the veterinarian to diagnose the disease, the symptoms not being as characteristic as in man. Generally among cattle the disease is in the last stage before known (Gurlit and Hanbuer). A correct report of the diseased animals cannot be obtained, therefore I will give some symptoms produced by feeding trichinæ, according to Gerlach's report, who thus far has had the best results. The symptoms both in man and animal do not differ much. Reports of producing trichinosis in animals we did not have until the beginning of the eighth decade from experiments made by Gerlach, Gurlt, and Hanbuer, who fed forty hogs of different ages with food containing trichinæ. This experiment showed that all ages are subject to the disease; in fact, the younger have it more severely than the older. The opposite is true in man—children with gastric symptoms being in less danger, making a quicker recovery than adults, although this may be due to having partaken of less food containing trichinæ. Trichinosis can be divided into two stages, intestinal and muscle trichinosis. The first stage diminished, sometimes complete loss of appetite, followed by general signs of sickness. Very soon symptoms with diarrhœa appear, which often causes death in small animals; generally a very marked weakness exists, wanting to rest continually. After ten or twelve days, until the third week, the intestinal trouble disappears. Muscular affections, resembling rheumatism, then appear, with symptoms similar to typhoid fever; stiffness, difficult swallowing, trismus, resembling a case of tetanus; inflammation of the eyes, œdematous swelling of the legs (but not as in man, œdematous swelling of the eyelids), and general marasmus. If the animal recovers all these symptoms quickly disappear. The greater the number of trichinæ the more pronounced and severe the symptoms. Virchow and Pagenstecher claim that the symptoms are not so characteristic as given by Gerlach. The disease is often fatal. If, after thorough examination, suspicion arises that the animal is infected, microscopic examination must be made, removing a small piece of muscle by means of a muscle-harpoon specially made for this purpose. The autopsy is of great importance, and it is easy to detect trichinosis by the œdematous swelling, complete flexion of legs, and general marasmus. In the muscles are found yellowish spots, the size of a pinhead, more or less, according to the location of trichinæ. Parenchymatous swelling is found on the intestinal organs, heart, liver, and kidneys. Very often bloody extravasations are found on the peritoneum, and sometimes, also, on the intestinal tissues. The appearance of the muscle is changed, losing through the existence of trichinæ the striated consistence, and becoming spotty and lighter. Leukart claims that the muscles become dark red; this may be so in the beginning, but not in old cases, when the muscles change to a grayish red, also to grayish yellow (Eichhorst).

*Prophylaxis.* Exposure of all pork before eating to high temperature and the use of salt. The first and most important rule is, never to eat raw or partly boiled pork. It should never be eaten in any form whatever unless thoroughly boiled, as the worm resists a very high temperature. The worm is not destroyed by smoking or pickling unless rent into small pieces and adding water (30 gr. sulph. of soda to 1000 gr. meat). Weaker pickling drawing less water and without energetic smoking has no effect whatever; likewise ham and sausage put in *Acetum lignorum.* Salt and smoke used properly certainly are a good preventive (Wolff, John, Tieman).

*b. Microscopic Examination.* For protection every hog slaughtered should be microscopically examined. The pieces for preparation should be taken from different parts, especially where trichinæ generally locate, for instance, the diaphragm, intercostal, throat, or jaw muscle, also where the muscles pass into the tendons where trichinæ are most frequently found. Small pieces of these different muscles should be taken for examination. If no trichnæ are found the pork is free from the worm and not dangerous to human life. Trichnæ to be seen well should be examined with an objective enlarging 40 to 50 diameters.

*c. Slaughtering in Abattoirs.* The experience in different countries shows the necessity of meat-inspection, and for thorough inspection the building of abattoirs. Recalling the different epidemics of trichinosis in 1865, in Hedersleben, Thuringia, a town of 2000 inhabitants, 33 per cent. took sick and 101 died

(Krate). In Hanover, during 1864 and 1865, three epidemics prevailed, causing the illness of 300 people. Since 1866 they have had meat-inspection, since then they experienced one epidemic, caused by using pork of one hog that had not been examined (Gerlach). The report by Blasius in Brunswick, in 1882, states that 254 people became sick with trichinosis. Bronardel reports 250 cases in Halberstadt in 1883. Riedel reports 255 cases in Obercuncwalde in 1888. Since then in all larger towns they have (Europe) abattoirs where meat is examined before being placed in the market, and a trichinæ epidemic is seldom heard of. My opinion is that it certainly is wrong and unjust to make a law to have all meat to be exported examined, but not that for the interstate trade. Live-stock inspection by the United States Government, Section 18, reads as follows: "A microscopic examination for trichinosis must be required for all swine-products exported to countries requiring examinations. No microscopic examination will be made of hogs slaughtered for interstate trade, but this examination will be confined to those intended for the export trade, and only at abattoirs which export pork-products to countries requiring a certificate from this government to secure the admission of such meats." To this law, remarks the State Board of Health of Pennsylvania, "the fact is universally known that for a considerable period several of the governments of Europe prohibited the importation of American pork for the alleged reason that much of it was infected with these parasites. In order to obtain the withdrawal of this prohibition the United States Government established a systematic inspection of all pork intended for export to those countries. There is good reason to suppose that about 3 per cent. of the hogs thus examined are condemned. There is no such protection afforded to American consumers, and there is room for apprehension that much even of this condemned trichinous pork, known to be such, finds its way into the home market. Only the honesty of dealers prevents this, and that is unfortunately a broken reed to lean upon." If we add to it the percentage of trichinous American pork, it is, according to Eulenburg, 4 per cent., Billings, 5.7 per cent., we will find the beforementioned remarks of the Board of Health perfectly right, and put to it the reports of trichinosis of the different States from where I got them.

Ontario: No case of trichinosis recorded during the last five years.

Quebec: No statistics thereon which an opinion can be based on the prevalence, or not, of trichinosis in this province.

Manitoba: There has never been a case of trichinosis reported to the Department of Health and therefore no statistics on this subject.

Maine: About six or eight years ago two boys, when butchering hogs on a farm, cut a small piece of pork and ate it. Both boys were attacked with trichinosis, but both recovered. The pork was examined by the State Board of Health and trichinæ were found.

Massachusetts: During the past ten years slight outbreaks of this disease have occurred, in which one or more persons, and in some cases one or two families, have been involved; but no fatal cases had occurred for more than twenty years until February, 1892, when notice was sent to the Board of existence of an epidemic of this character of somewhat alarming extent in the town of Colrain, Franklin County. Here was an epidemic of more than fifty cases, with four deaths, witnessed by three physicians, yet it was not until the second death, and three or four weeks from its commencement, that a correct diagnosis was reached; and it is possible that if there had been no fatal cases the diagnosis of trichinosis might never have been made. The cause was Chicago bologna-sausage. Soon after the occurrence of this outbreak several other cases were reported from Roxbury, with one death. Some of the cases were reported by Dr. C. W. McDonald. The cause of illness in these cases was undoubtedly ham eaten raw or partially cooked. About fifteen persons were afflicted, with one death. As Secretary of Agriculture Rusk has established an official laboratory in Chicago for the examination of pork intended for export a larger proportion of trichinous meat will be sold in the American market and the disease will be likely to prevail to a greater extent than heretofore. What becomes of the pork condemned as unfit to be exported? It is worked into these cheap sausages, which were probably the cause of this epidemic. Figures show that 16 per cent. of American pork is trichinosed (A. Leibert, New York).

Rhode Island: No report was made on this subject. By reference to mortality statistics for the full period of registration of eighteen or nineteen years, only one death recorded from that cause. This death occurred in 1893. Four in one family were affected. The material ingested was fat pork, bought from a travelling butcher-cart.

New York: Only one outbreak in the past fifteen years in Arietta, Hamilton County. On the 2d of July, 1884, Dr. J. E. Burchick, of Johnstown, stated to the Board of Health that he had in charge several cases of fever of some kind at Arietta, and that there was some doubt regarding the nature of the sickness. Microscopic examination showed trichinæ. Twelve cases of infection, with two deaths. Cause: Raw ham from Chicago.

New Jersey: The Secretary of the State Board of Health states that no separate record of this disease is kept, and that he can remember but few cases for several years.

Pennsylvania, 1885 to 1895: To give an idea of the frequency of this disease it may be mentioned that in the space of ten years the former President of the State Board of Health, Dr. Ed. H. Germer, in his capacity as Health Officer of Erie, detected no less than six instances in and near that city causing several deaths and not less than fifty cases of serious and painful illness. There is no good reason for supposing that it is more frequent in that part of the State than in any other, but its presence constantly escapes recognition. In 1886, during the epidemic at Bethlehem, one death occurred. The Board is at present investigating two deaths in the city of Lancaster recently, supposed to be due to this cause. One case occurred in Reading during the month of August.

Ohio: No report of trichinosis has been issued in this State, but some outbreaks in this State I know from newspaper reports. The last occurred in April, 1895, at Toledo. Of five sick in one family, three died. Microscopic examination showed trichinæ in corpses and meat. Meat came from Chicago.

Indiana: No report of trichinosis there.

Delaware: The State Board of Health did not have such reports.

Tennessee: Only one authentic report of trichinosis made to the Board has occurred in the last ten years. Reported by Dr. McCall Huntingdon, one family of seven persons affected in December, 1885. Cause: Eating the flesh of home-raised meat.

West Virginia: No reports of trichinosis. Secretary is not aware of any death from the disease in this State for years.

Missouri: No reports there.

North Carolina: No reports.

Alabama: Secretary writes that he never heard of a case of trichinosis in Alabama.

Minnesota: No outbreaks have occurred.

Michigan: No outbreaks of trichinosis since December, 1866. A fatal case of trichinosis occurred in Detroit in December, 1866. Five cases occurred in Port Huron in January, 1874, with two deaths, caused by eating salted smoked ham. Several cases of trichinosis occurred near Flint in the fall of 1875. Cases also occurred there in June, 1876. Five cases occurred in Otsego in February, 1877. In one family the members had eaten raw ham. Members of another family ate a small quantity of the meat and were also sick, but recovered without medical attendance. Three cases with one death in Jonia, 1873. Cases with one or more deaths occurred in the vicinity of Jonia in the summer of 1880. Five cases and two deaths in Lansing in January, 1887. Six cases in Virkyville, with one death, in December, 1883. Remarkable in this case that the meat was well cooked. Four cases in January, 1884, in Niles; one death. Four cases in July, 1884, in Cassopolis; one death (rats fed to the hogs). In all cases microscopical examination showed trichinæ.

Our duty, gentlemen, I think, is to work for to complete this law that the lives of our citizens get more protection.

